# Testicular fusocellular rhabdomyosarcoma as a metastasis of elbow sclerosing rhabdomyosarcoma: A clinicopathologic, immunohistochemical and molecular study of one case

**DOI:** 10.1186/1746-1596-5-52

**Published:** 2010-08-11

**Authors:** Miguel Martorell, Cristian M Ortiz, Jose Angel Garcia

**Affiliations:** 1 Department of Pathology, Hospital General Universitario de Valencia, University of Valencia, Spain

## Abstract

Sclerosing rhabdomyosarcoma (SRMS) is an infrequent variant of rhabdomyosarcoma characterized by extensive intercellular hyaline fibrosis. We report the case of a 37 year-old male with a 9 × 6 cm SRMS on the right elbow. Histologically, the tumor showed an abundant extracellular hyaline matrix with extratumoral vascular emboli and microscopic foci of fusocellular embryonal rhabdomyosarcoma (FRMS) separated by a fibrotic band from the sclerosing areas. One year later the patient presented with a right intratesticular tumor of 1.2 × 0.8 cm, which was reported as pure FRMS. Immunohistochemically, SRMS was positive only for MyoD1 and Vimentin and negative for Myogenin and Desmin. Both the elbow emboli with the extratumoral foci of FRMS and the intratesticular tumor were positive for Myogenin, MyoD1, Vimentin and Desmin. Using fluorescent *in situ *hybridization (FISH), the SRMS and the FRMS tumor cells of the elbow and the FRMS tumor cells of the testis were found to be negative for FOXO1A translocation in chromosome 13. PCR chimeric transcriptional products PAX3-FKHR and PAX7-FKHR were not found. Six months following testicular resection, the patient died of multiple metastases in the mediastinum, lung and right thigh.

## Background

Rhabdomyosarcoma (RMS) is the most common sarcoma developed during childhood. It is a devastating tumor displaying characteristics of muscle differentiation. Rhabdomyosarcoma currently is classified into three main groups: (i) embryonal (ERMS), (ii) alveolar (ARMS) and (iii) pleomorphic (PRMS), each of which can show several significant clinical, morphological, molecular and prognostic differences. ERMS tumor cells, including its variants, botryoid, anaplastic and spindle or fusocellular cells (FRMS), often occur in infants and adults [1]. In non-pediatric cases embryonal rhabdomyosarcoma usually appears in the head, neck and extremities, and does not contain PAX3/FOXO1A or PAX7/FOXO1A fusion proteins, expressing clearly all muscle immunohistochemical markers. The sclerosing variant of rhabdomyosarcomas (SRMS) was first described by Mentzel and Katenkamp [2]; 16 cases in adults have been reported [2-8], all of which share the particular characteristic of diffuse hyaline fibrosis surrounding the sarcomatoid cells. Although sclerosing rhabdomyosarcoma has not yet been classified among the different types of RMS, some authors consider that it could be the same neoplasm [9] or a subtype of fusocellular rhabdomyosarcoma on the basis of histological, immunohistochemical and chromosomal changes [3]. We present a case of sclerosing rhabdomyosarcoma of elbow showing extratumoral intravascular emboli and stromal external foci of fusocellular rhabdomyosarcoma, histologically and immunohistochemically identical to the metastasis that appeared one year later in the right testis. In our opinion this case supports a close histogenic relationship between sclerosing and fusocellular rhabdomyosarcoma, given that we could not exclude sclerosing rhabdomyosarcoma as representing a histological type of fusocellular rhabdomyosarcoma.

## Case presentation

**Figure 1:**
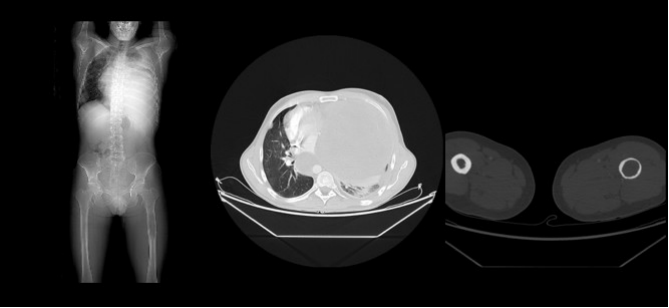
**CT Scan showing tumor progression**. Eight months after testicular resection, the left lung was completely occupied by a metastatic tumor mass involving mediastinum. The left thigh was also affected by the metastases.

A 37 year-old male without significant previous medical history, presented in June 2007 with a solitary soft tissue mass in the posterior side of the right elbow, with rapid growth that did not infiltrate the bone or the skin. The patient underwent a tumorectomy with wide margins. Following histological study, he received three cycles of chemotherapy (CTX+VCR+Doxorrubicin). One year after tumorectomy, a "de novo" intratesticular tumor appeared in the right testis, not previously detected. Orchiectomy was subsequently performed. A second line of chemotherapy (Ifosfamide) followed, but without positive response. Eight months following the orchiectomy, the patient died with multiple metastases in the left thigh, lung and mediastinum. No autopsy was performed (Figure 1)

The tumors were processed in molecular fixative solution for Tissue-Tek Xpress Rapid Tissue Processor^® ^(Sakura), embedded in paraffin, cut in 4 μm thick sections and stained with hematoxylin and eosin for routine histological examination. Immunohistochemical analysis was done on the paraffin-embedded sections using standard protocol. Primary antibodies were: MyoD1 (Dako, diluted 1:50), Vimentin (Dako, diluted 1:100), Myogenin (Dako, diluted 1:50), SMA (specific muscle actin) (Dako, diluted 1:100), Desmin (Dako, diluted 1:100), EMA (Dako, Diluted 1:50), CD 99 (Signet Laboratories, Dehman, MA, USA, diluted 1:50), p57 (Dako, diluted 1:200), CD 56 (Dako, diluted 1:200), S100 protein (Dako, diluted 1:100), CEAp (Dako, diluted 1:400), CD31 (Dako, diluted 1:1000), CD34 (Dako, diluted 1:1000), MIB 1 (Dako, diluted 1:400), and pan-cytokeratin (AE1/AE3, Dako Glostrup, Denmark, diluted 1:50). Fluorescence *in situ *hybridization (FISH) and RT-PCR were carried out on the paraffin-embedded sections from the elbow and testicle tumors. For FISH we used the Poseidon Repeat Free FKHR (13q14) protocol (Kreatech Diagnostics, Amsterdam, the Netherlands). The probe used was FKHR (FOXO1A, 13q14) Break apart (POSEIDON). There are two critical regions: (i) critical region 1 (red), in which the distal FKHR gene region probe was directly labeled with Platinum Bright 550; and (ii) critical region 2 (green), in which the proximal FKHR gene region probe was directly labeled with Platinum Bright 495. Analysis was performed with a fluorescent microscope, and the software used for image interpretation was CW4000. RT-PCR was performed as previously reported [4], using adequate primers to detect the chimeric transcriptional products PAX3-FKHR and PAX7-FKHR.

Grossly the surgical specimen from the elbow showed a 9 × 6 cms nodular solid tumor, white in color with a glistening surface, infiltrating the surrounding skeletal muscle.

**Figure 2:**
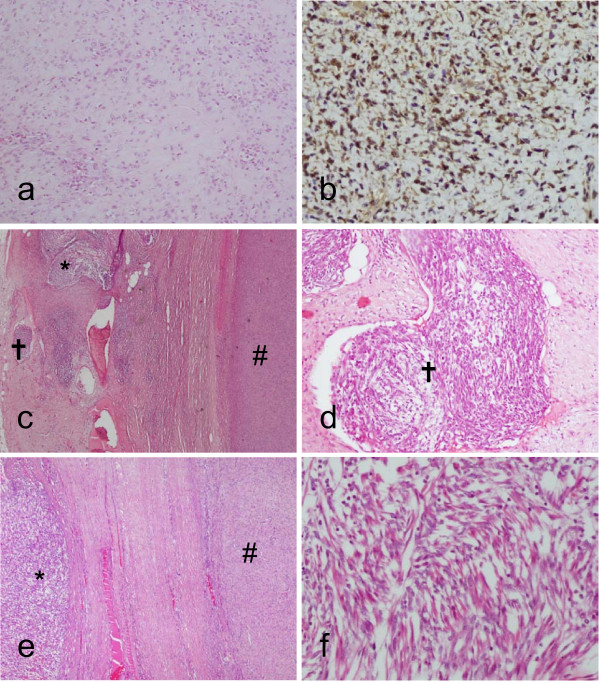
**Histological features from the surgical specimen from the elbow**. a: Tumor with abundant esosinophilic hyalinizing sclerotic matrix (10×). b: Immunohistochemical staining positive for MyoD1 (20×). c: Sclerosing area (#) with fusocellular foci (*) and vascular emboli (✝) of FRMS (10×). d: Vascular emboli of FRMS (20×). e: Sclerosing rhabdomyosarcoma separated from FRMS foci by a fibrotic band (10×). f: FRMS foci with spindle-shaped cells and rhabdomyoblastic differentiation (40x).

Histologically, the tumor had abundant esosinophilic hyalinizing sclerotic matrix, containing predominant small round cells growing in small nests of fascicular, pseudovascular cord-like or single file. In some areas a predominant perivascular tumor growth was observed. No microalveolar pattern was seen. Wreath-like tumor cells and strap rhabdomyoblasts were not identified. Mitotic activity was up to 4 mitotic figures/10 HPF. Coagulative necrosis occupied 10% of the tumor mass. On hematoxylin and eosin findings an initial diagnosis of sclerosing sarcoma, most probably epitheloid sclerosing fibrosarcoma was reached (Figure 2).

Immunohistochemically the tumor cells strongly expressed Vimentin, MyoD1, SMA and CD99, being negative for the remaining antibodies tested, including Myogenin and Desmin. The proliferation index measured with MIB 1 was about more than 25%-30% of tumor cells.

**Figure 3:**
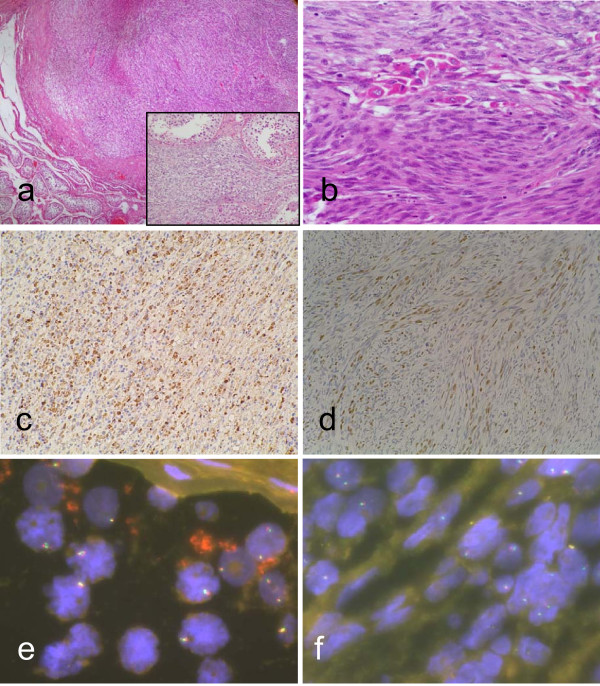
**Histological and molecular features of testicular FRMS**. a: Peripheral FRMS subalbuginea intratesticular well-defined nodular tumor (10×), infiltrating the surrounding testicular parenchyma (insert) (20×). b: Fascicles of spindle-shaped cells with small oval or elongated nuclei, vesicular chromatin and pale cytoplasm accompanied by rhabdomyoblastic differentiation (40×). c and d: Immunohistochemical staining was positive for MyoD1 and Myogenin. E: FISH hybridization negative FOXO1A: 13q14 translocation, in the elbow sclerosing rhabdomyosarcoma. f: FISH hybridization did not show FOXO1A: 13q14 translocation, in the FRMS.

FISH hybridization did not show FOXO1A: 13q14 translocation and the chimeric transcriptional products PAX3-FKHR and PAX7-FKHR were also not found (Figure 3).

On gross examination the orchiectomy specimen showed a peripheral subalbuginea intratesticular well-defined nodular tumor of 1.2 × 0.8 cms, grey to white with solid consistency, with central necrosis and delimited by a fibrous capsule from the normal testicular parenchyma. Histologically the tumor showed features of fusocellular rhabdomyosarcoma, composed of fascicles of spindle-shaped cells with small oval or elongated nuclei, vesicular chromatin and pale cytoplasm. Occasionally scattered in the tumor were a small number of spindled or polygonal cells with excentrically placed nuclei and abundant eosinophilic cytoplasm, resembling more mature rhabdomyoblasts, were also seen. Mitotic activity was more than 10 mitotic figures/10 HPF. The tumor infiltrated the surrounding testicular parenchyma and was centrally necrosed (30%). Immunohistochemically the cells stained positively for Vimentin, MyoD1, CD99, Myogenin, Desmin, SMA and WT-1. The proliferation index measured with MIB was about 20%. Again, FISH failed to show the FOXO1A: 13q14 translocation and the chimeric transcriptional products PAX3-FKHR and PAX7-FKHR were not found.

After the histological diagnosis of the surgical specimen from the testicular lesion, a complete sampling of the sclerosing rhabdomyosarcoma from the elbow treated one year before was performed. An extensive histological study of the new samples demonstrated new histological findings, such as the presence of two large endovascular (venous) emboli and 3 minute foci of tumor (measuring all together less than 3 mm) located in the non-tumoral soft tissue, next to the sclerosing rhabdomyosarcoma and apparently isolated from them by a fibrotic acellular band. Histologically, tumor cells from the emboli and extratumoral foci showed predominant fusocellular morphology. A few isolated round primitive cells without sclerosis and strap rhabdomyoblastic cells were also present. Immunohistochemically these cells expressed Vimentin, MyoD1, Myogenin, CD 99, SMA and Desmin, being histologically and immunohistologically identical to the testicular tumor studied one year later (Table 1 and Figure 3).

**Table 1:** Clinicopathological findings. Chronology

CLINIPATHOLOGYCAL FINDINGS - CHRONOLOGY			
DATE	June 2007	July 2008	February 2009

LOCATION	Mass in the right elbow	Intratesticular tumor	Patient died with multiple metastases in the left thigh, lung and mediastinum. No autopsy was performed
	
HISTOLOGY	Eosinophilic hyalinizing sclerosing matrix with small round cells	Fusocellular RMS with eosinophilic matrix and rhabdomyoblasts	
	
MITOSIS	4 mit/10HPF	10mit/10HPF	
	
IHC (+)	Vimentin	Vimentin	
	MyoD1	MyoD1	
	SMA	SMA	
	CD99	CD99	
		myogenin	
	
MIB-1	25-30%	20%	
	
RT-PCR	PAX3-FKHR and PAX7-FKHR were not found.	PAX3-FKHR and PAX7-FKHR were not found.	
	
FISH	FOXO1A (-)	FOXO1A (-)	
	
Chemotherapy	CTX+VCR+Doxorrubicin	Ifosfamide	
	
Surgery	Tumor resection	Right Orquidectomy	

## Discussion

We have presented a unique case of a 37 year-old male diagnosed with sclerosing rhabdomyosarcoma in the elbow containing vascular emboli and peritumoral microscopic foci of fusocellular rhabdomyosarcoma. He developed a pure intratesticular fusocellular rhabdomyosarcoma one year after the initial diagnosis.

Sclerosing rhabdomyosarcoma is an infrequent tumor. In adults only 16 cases have been reported. These bearing a slight male predominance: the overall median age at diagnosis was 46.6 years (range 18-79 years), and most frequently appeared as tumors of the extremities as well as the head and neck region [5-7]; their prognoses appeared worse than previously reported [7]. Among the cases with available follow-up reported, four presented metastasis or died between 7 to 48 months after diagnosis [5,7,10]; six had recurrences or residual tumor [2,5,6,8,9], and only 4 patients were free of disease in a follow up between 5 and 26 months [2,7,11].

As initially described by Mentzel and Katenkamp in 2000 [2] sclerosing rhabdomyosarcoma is characterized by the production of prominent extracellular diffuse hyaline sclerosis, with different growth patterns which can may mimic sclerosing epitheloid fibrosarcoma, osteosarcoma, chondrosarcoma or angiosarcoma.

Immunohistochemically sclerosing rhabdomyosarcoma in adults shows a distinctive immunophenotype in the expression of muscle markers, characterized by positivity for MyoD1, negativity for Myogenin and variable expression for Desmin and Actin muscle specific [3,7], as occurred in the elbow tumor. Contrarily, pediatric cases of sclerosing rhabdomyosarcoma expressed strong immunopositivity for all muscle markers, including MyoD1, Myogenin, Desmin and SMA, most likely reflecting a different stage of muscle maturity [12].

The histogenic relationship between sclerosing rhabdomyosarcoma and the other types of rhabdomyosarcomas remains unclear although cytogenetic studies have suggested a link with embryonal rhabdomyosarcoma [11,13,14].

Previous histological studies have pointed-out that sclerosing rhabdomyosarcoma may be closely related to embryonal rhabdomyosarcoma. Intratumoral small foci of rhabdomyoblastic strap cells have been observed in cases of sclerosing rhabdomyosarcoma [5,7,14]. The most common phenomenon is the presence of a spindle cell component, closely similar to fusocellular rhabdomyosarcoma, a subtype of embryonal rhabdomyosarcoma. In fact, almost a third of the sclerosing rhabdomyosarcomas reported in adults (5 cases) presented intratumoral microscopical foci of rhabdomyoblastic or spindle cell differentiation [2,3,5-7]. In addition there are several adult cases of fusocellular rhabdomyosarcoma containing areas of focal [10] or extensive hyaline sclerosis [15].

In the present case we also observed spindle and rhabdomyoblastic differentiation, but our findings have two main differences with other cases previously reported. First, spindle or rhabdomyoblastic differentiation appeared only in extratumoral areas infiltrating preexisting normal tissue or embolizing peritumoral vessels. No fusocellular or rhabdomyoblastic differentiation was identified in the sclerosing rhabdomyosarcoma. Second, and the more important difference, the peritumoral and intravascular infiltrating cells growing in a predominant spindle cell pattern showed a similar histological, immunohistochemical and molecular profile than that of the cells from the testicular fusocellular rhabdomyosarcoma diagnosed one year latter.

Fusocellular rhabdomyosarcoma is a type of embryonal rhabdomyosarcoma that was described by Mentzel et al. in 2006 [10]. Histologically, the tumor is made up composed of atypical spindle-shaped tumor cells arranged in cellular fascicles with small, oval to elongated and ill-defined nuclei, small nucleoli and pale indistinct cytoplasm. Occasionally, the tumor cells have a varying number of rhabdomyoblasts containing abundant eosinophilic cytoplasm as well as hyperchromatic and excentrically placed enlarged nuclei. The identification of this second cell type is a distinct indicator for the diagnosis of fusocellular rhabdomyosarcoma [16]. Fusocellular rhabdomyosarcoma in adults arises most frequently in the extremities as well as head and neck, and is characterized clinically by poor prognosis, as opposed to fusocellular rhabdomyosarcoma in children and adolescents, which is characterized by paratesticular location and excellent clinical prognosis [10].

Primary testicular spindle cell rhabdomyosarcoma is extremely infrequent in adults; only isolated cases have being described in the paratesticular region [17,18].

On the other hand, testicular metastasis from rhabdomyosarcoma is a very rare phenomenon, representing approximately only 1% of testicular tumors [18,19]. Only four pediatric cases have been reported worldwide [18-21], all corresponding to a metastasis from alveolar rhabdomyosarcoma. In all the cases, metastatic tumors conserved the same histological and immunohistochemical phenotype of the primary tumor.

## Conclusions

If our case follows this rule, it further supports that testicular spindle cell rhabdomyosarcoma represents a metastases from the cells with spindle or rhabdomyoblastic differentiation infiltrating vessels or soft tissue observed in the peritumoral vicinity of the sclerosing rhabdomyosarcoma of the elbow. In our opinion, the reported case highlights the close relationship between fusocellular rhabdomyosarcoma and sclerosing rhabdomyosarcoma, supporting the hypothesis (at least in adults) that most probably sclerosing rhabdomyosarcoma represents a subtype or type of fusocellular rhabdomyosarcoma sharing a common origin.

## Competing interests

The authors declare that they have no competing interests.

## Authors' contributions

MM and CO conceived of the study and participated in the design of the manuscript and performed the histopathological examination. JAG carried out the molecular genetic studies and drafted the manuscript. All authors have read and approved the final manuscript.
